# Patient outcomes following GPs’ educations about COPD: a cluster randomized controlled trial

**DOI:** 10.1038/s41533-020-00204-w

**Published:** 2020-10-15

**Authors:** Hanna Sandelowsky, Ingvar Krakau, Sonja Modin, Björn Ställberg, Sven-Erik Johansson, Anna Nager

**Affiliations:** 1 Karolinska Institutet, Department of Medicine, Division of Clinical Epidemiology, SE-171 76 Stockholm, Sweden; 2 Academic Primary Health Care Centre, Stockholm County Council, Box 45436, SE-104 31 Stockholm, Sweden; 3 Karolinska Institutet, NVS, Section for Family Medicine and Primary Care, Alfred Nobels Allé 23, Huddinge, SE-141 83 Stockholm, Sweden; 4 Uppsala University, Department of Public Health and Caring Sciences, Family Medicine and Preventive Medicine, Box 564, SE-751 22 Uppsala, Sweden; 5 Lund University, Center for Primary Health Care Research, Department of Clinical Sciences, SE-205 02 Malmö, Sweden

**Keywords:** Chronic obstructive pulmonary disease, Health occupations

## Abstract

This study aimed to compare patient outcomes following case method learning and traditional lectures as methods for continuing medical education (CME) about chronic obstructive pulmonary disease (COPD) for general practitioners (GPs) in Sweden. In a pragmatic cluster randomized controlled trial, COPD patients (*n* = 425; case method group *n* = 209, traditional lectures group *n* = 216) from 24 primary health care centers replied to questionnaires prior to and 18 months after a 2 × 2-h CME was given to GPs (*n* = 255). We measured changes in the scores of the Clinical COPD Questionnaire (CCQ), symptoms, needs for disease information, exacerbations, smoking, and use of pulmonary rehabilitation. The changes over time were similar for both CME methods. Patients who had used pulmonary rehabilitation increased from 13.2 to 17.8% (*P* = 0.04), and prevalence of smoking decreased from 28.9 to 25.1% (*P* = 0.003). In conclusion, neither of the used CME methods was superior than the other regarding patient outcomes. CME’s primary value may lay in improving GPs’ adherence to guidelines, which should lead to long-term positive changes in patient health.

## Introduction

Chronic obstructive pulmonary disease (COPD) is a public health burden that causes suffering and mortality. The Global Initiative for Obstructive Lung Disease (GOLD) guidelines aim to improve quality of life and prognosis by recommending treatments to reduce symptoms and prevent exacerbations^1^. The current treatment recommendations for patients with moderate to severe COPD are based on both non-pharmacological treatments (e.g., smoking cessation, pulmonary rehabilitation, and nutritional therapy) and pharmacological treatments. Assessments of disease progress and therapeutic choices are mainly made by monitoring the development of symptoms and exacerbations. Optimal COPD care can best be delivered via person-centered care given by interprofessional teams. In Sweden, the majority of patients with COPD are managed in primary health care. However, as elsewhere, there is a continuing need for improvements in general practitioners’ (GPs) adherence to guidelines^2^.

Continuing medical education (CME) is commonly used to improve guideline implementation in clinical practice. Two examples of short educational outreach visits that are popular among GPs are participatory case method learning^3^ and didactic, traditional lectures. However, educational researchers have questioned the effectiveness of didactic teaching methods^4^. Moreover, research in Swedish primary health care shows that case-based training is associated with decreased mortality in patients with coronary heart disease^5^. This evidence about teaching methods led us to design and conduct PRIMAIR, a pragmatic cluster randomized controlled trial to improve COPD guideline adherence in GPs in Stockholm, Sweden, using and comparing two CME methods^6^. The first published results of PRIMAIR described the educational outcomes in GPs. We observed that both case method learning and traditional lectures led to modest, but equally significant improvements in GPs’ levels of knowledge about COPD^7^. To gain information about whether GPs’ CME improved the management of COPD and patients’ health status, this part of PRIMAIR included an assessment of clinical (patient) outcomes. We hypothesized that case method learning would be better than traditional lectures at improving health-related outcomes in patients with COPD.

This study aimed to compare and describe the effects of two educational methods used for GPs’ education in COPD, regarding patient outcomes including health status, symptoms, patients’ perceived information needs about COPD, exacerbations, smoking, and health-care visits.

## Results

### Description of the patients

**Fig. 1:**
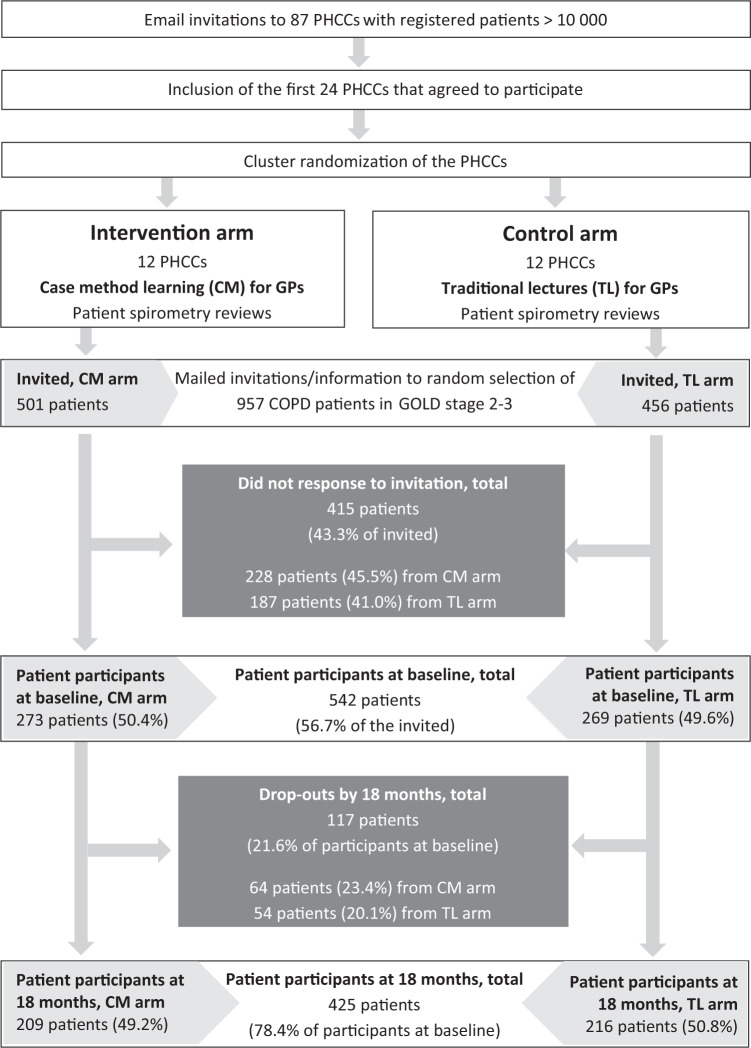
Trial enrollment flow chart. PHCC primary health care center.

At baseline, 542 patients completed the questionnaire (response rate 57%), and at 18 months, 425 replied to the follow-up questionnaire (final response rate 44%) (Fig. 1). The non-responders at baseline were slightly younger than the responders (mean age 70.3 vs. 72.0 years, *P* = 0.01) and more of them were in GOLD stage 2 (68% vs. 57%, *P* = 0.001). The drop-out rate between baseline and 18 months was independent of CME arm, patient’s age, gender, lung function, exacerbation rate, health status (scores in Clinical COPD Questionnaire (CCQ), COPD Assessment Test (CAT), and modified Medical Research Council dyspnea scale (mMRC), the body mass index, smoking status, level of education, and whether the patient was living alone or not.

There were no important differences in the baseline characteristics of the patients in the two study arms (Table 1). A higher percentage of patients in the case method learning arm than the traditional lecture arm attended a primary health care center (PHCC) with a nurse-led asthma/COPD clinic (62% vs 49%, *P* = 0.002).

**Table 1:** Patient characteristics at baseline (*n* = 425).

Characteristics, *n* (%)	All patients, *n* = 425 (100.0)	Case, *n* = 209 (49.2)	Traditional, *n* = 216 (50.8)	*P* value
Age				
Years, mean [95% CI]	71.8 [71.0–72.6]	72.6 [71.4–73.7]	71.0 [69.9–72.1]	n.s.
Age distribution, *n* (%), years				
Age 35–64	73 (17.2)	26 (12.4)	47 (21.8)	0.014
Age 65–79	267 (62.8)	134 (64.1)	133 (61.6)	n.s.
Age 80–93	85 (20.0)	49 (23.4)	36 (16.7)	n.s
Gender				
Female, *n* (%)	267 (62.8)	128 (61.2)	120 (55.6)	n.s.
Education				
≤9 years, *n* (%)	221 (54.6) (missing data = 20)	104 (52.8)	117 (56.3)	n.s.
Smoking intensity				
Pack years, mean [95% CI]	33.6 [31.5–35.6] (missing data = 74)	33.6 [30.4–35.1]	33.5 [30.4–35.8]	n.s.
FEV1 after bronchodilator				
% of predicted value	56.2 [54.8–57.5]	56.6 [54.7–58.6]	55.7 [53.8–57.6]	n.s.

None of the patient outcomes at 18 months differed between the arms (Table 2). However, within each arm, there were significant changes over time in several of the outcomes (Table 3).

**Table 2:** Patient outcomes as difference in differences (DiD) between baseline and at 18 months comparing case method learning to traditional lectures and expressing treatment effect as (Δ score and Δ% units) (*n* = 425).

Patient outcomes	Educational method					
	Case method learning		Traditional lectures		Difference in differences (DiD)	
	Baseline	18 months	Baseline	18 months		
	Mean (95% CI)	Mean (95% CI)	Mean (95% CI)	Mean (95% CI)	Δ score^a^	*P* value (95% CI)
CCQ^b^	1.84 (1.68–2.00)	1.97 (1.81–2.14)	1.89 (1.73–2.06)	1.97 (1.81–2.13)	0.05	0.64 (−0.16 to 0.26)
CAT^c^	14.7 (13.6–15.8)	16.2 (15.1–17.4)	15.5 (14.4–16.6)	16.3 (15.2–17.5)	0.67	0.21 (−0.37 to 1.72)
LINQ^d^	11.0 (10.5–11.6)	10.7 (10.1–11.3)	10.8 (10.3–11.3)	10.6 (10.1–11.1)	−0.19	0.71 (−1.16 to 0.78)

	Baseline %	18 months %	Baseline %	18 months %	Δ% units^e^	*P* value (95% CI)
Current smokers	29.7	27.8	28.2	22.4	3.3	0.15 (−1.2 to 7.9)
mMRC^f^ ≥ 2 points	43.9	56.1	43.4	50.0	5.3	0.34 (−5.5 to 16.1)
Exacerbations (one or more, past 6 months)	33.5	34.7	33.3	34.1	0.4	0.93 (−9.9 to 10.7)
Influenza vaccination	69.3	70.7	65.5	67.9	−1.2	0.76 (−8.9 to 6.5)
Hospital admissions (one or more, past 6 months)	5.3	8.3	5.6	8.8	−0.4	0.90 (−6.5 to 5.7)
Assigned general practitioner	71.6	64.0	72.1	61.5	3.4	0.51 (−6.8 to 13.6)
Visited COPD nurse (past 12 months)	36.5	36.2	37.5	41.0	−3.4	0.50 (−16.8–10.0)
Visited physiotherapist (past 12 months)	10.6	16.8	15.7	18.8	3.0	0.49 (−5.5 to 11.6)
Visited occupational therapist (past 12 months)	2.9	6.1	4.2	5.6	1.8	0.52 (−3.6 to 7.2)
Visited nutritionist (past 12 months)	3.4	8.1	9.7	9.4	5.1	0.18 (−2.3 to 12.5)

*CI* confidence interval.

^a^Δ scores = [difference (18 months − baseline) in scores in case method learning arm] – [difference in scores in traditional lectures arm]. Please see the “Statistics” section for an explanation of the calculations.

^b^The Clinical COPD Questionnaire.

^c^The COPD Assessment Test.

^d^The Lung Information Needs Questionnaire.

^e^Δ% units = [Δ% units (18 months − baseline) in case method learning arm] − [Δ% units in traditional lectures arm]. Please see the “Statistics” section for an explanation of the calculations.

^f^The Modified Manchester Research Council Dyspnea Scale.

**Table 3:** Patient outcomes in the whole study population (*n* = 425) as difference in scores (%) between baseline and 18 months (Δ scores and Δ% units) when adjusted for clusters (primary health care centers) and for nurse-led COPD clinics.

Patient outcomes	Total (*n* = 425)		Difference over time	
	Baseline, mean (95% CI)	18 months, mean (95% CI)	Δ scores^a^	*P* value (95% CI)
CCQ^b^	1.87 (1.75–1.98)	1.97 (1.86–2.09)	0.12	0.03 (0.01–0.22)
CAT^c^	15.1 (14.3–15.9)	16.3 (15.5–17.1)	1.02	<0.001 (0.50–1.54)
LINQ^d^	10.9 (10.5–11.3)	10.6 (10.2–11.0)	−0.025	0.31 (−0.74–0.23)

	Baseline (%)	18 months (%)	Δ% units^e^	*P* value (95% CI)
Current smokers	28.9	25.1	−3.0	0.008 (−5.2 to −0.8)
mMRC^f^ ≥ 2 points	41.8	51.1	9.0	0.001 (3.5–14.6)
Exacerbations (one or more, past 6 months)	49.8	50.2	1.0	0.70 (−4.1 to 6.2)
Influenza vaccination	67.4	69.3	1.9	0.34 (−2.0 to 5.7)
Hospital admissions (one or more, past 6 months)	5.4	8.6	3.5	0.047 (0.04–6.2)
Assigned general practitioner	71.8	62.7	−9.3	<0.001 (−14.4 to −4.1)
Visited COPD nurse (past 12 months)	37.0	38.7	1.5	0.66 (−5.2 to 8.3)
Visited physiotherapis (past 12 months)	13.2	17.8	4.5	0.04 (−5.5 to 11.6)
Visited occupational therapist (past 12 months)	3.5	5.8	2.3	0.09 (−0.4 to 4.9)
Visited nutritionist (past 12 months)	6.6	8.7	2.1	0.28 (−1.6 to 5.8)

^a^Δ scores = scores at 18 months − scores at baseline, by the model, both study arms together. Please see the “Statistics” section for an explanation of the calculations.

^b^The Clinical COPD Questionnaire.

^c^The COPD Assessment Test.

^d^The Lung Information Needs Questionnaire.

^e^Δ% units = % at 18 months − % at baseline, by the model, both study arms together. Please see the “Statistics” section for an explanation of the calculations.

^f^The Modified Manchester Research Council Dyspnea Scale.

In the study group as a whole, the total scores of the CCQ^8^ and the CAT^9^, increased significantly over time from 1.87 to 1.97 and from 15.1 to 16.3, respectively, i.e., the health status slightly deteriorated over time. However, although statistically significant, the changes in the total scores were not clinically relevant^10,11^. The scores in the CCQ domains “symptoms” and “functionality” also increased between baseline and follow-up, whereas scores in the domain “mental health” remained unchanged. CCQ scores at 18 months were not associated with gender, age, or presence of cardiovascular comorbidity. However, patients who had anxiety/depression, obstructive sleep apnea, or chronic pain at baseline had clinically significantly higher CCQ scores at 18 months than patients who did not have these comorbidities.

The proportion of patients who had seen a physiotherapist increased over the study period (13.2–17.8%, *P* = 0.04). A significant percentage of the patients had stopped smoking; smoking prevalence decreased from 28.9% to 25.1% (*P* = 0.008). The percentage of smokers who had been offered smoking cessation support by a health-care professional increased from 51.2 to 61.9% (*P* = 0.038).

## Discussion

The 18-month follow-up of this pragmatic, real-life cluster randomized controlled trial of 542 COPD patients from 24 Swedish PHCCs did not support our hypothesis: short CME sessions for GPs using case method learning did not lead to greater improvements in patient-related health outcomes than did short traditional lectures. However, in the study population as a whole, the percentage of patients who had received pulmonary rehabilitation increased and the percentage of smokers decreased. These findings may have been an effect of changes in GPs’ management of COPD caused by meeting the intended learning outcomes (ILOs) of PRIMAIR’s CME sessions. Independent of the CME method, the significant changes in patients’ health status, symptoms, frequency of exacerbations, and hospital admissions indicated slight but general worsening of COPD.

In our study, traditional lectures, representing didactic CME methods, were as effective as the more participatory case method learning when applied at short sessions for GPs. However, results from previous research discourage CME educators from using didactic CME methods and rather suggest the use of multiple, participatory, educational activities as they have shown to enhance learning more than didactic educational methods^12,13^. As didactic CME is the type of CME GPs often prefer^14^, and as it is easy to conduct and apply in practice, we argue that these traditional CME lectures should not be overlooked as a useful learning method for GPs.

Evidence on the most effective and feasible types of CME is still incomplete^15^. Compared to educational and pedagogical research about undergraduate learning, CME research is a fairly young discipline. To contextualize the results of the current study, it is essential to understand the difficulties of studying the effects of single CME interventions. For example, individual CME interventions, like the one in in this study, do not generally result in large absolute effect sizes. On the basis of the statement of The Cochrane Effective Practice and Organisation of Care Group^16^, in 2012, Grimshaw et al. concluded that systematic reviews should be used to evaluate and develop CME programs. They reasoned that studies about individual CME interventions may be misleading because of bias in their conduct or random variations in their findings^15^.

At the 18-month follow-up, we found an increase in the percentage of patients who used pulmonary rehabilitation and decrease in percentage of current smokers. These findings may be related to disease progress, or GPs’ improved adherence to guidelines, or both. Other CME projects have led to increased use of pulmonary rehabilitation^17^, so we reason that the CME in in this study may have had similar effects. Previously published results on PRIMAIR’s baseline data showed that the participating GPs had a high level of knowledge about smoking cessation support^18^, yet they rarely offered smoking cessation support to patients^19^. The improvements in the level of smoking cessation support and the reduction in the number of patients who still smoked might thus have been effects of the CME. On the other hand, patients whose COPD continues to deteriorate, as in our study population, are often motivated to quit smoking^20^.

We observed a small deterioration in COPD measured as increased CCQ and CAT scores over the 18-month study period. Previously, the progression of COPD was determined by the decline in FEV1 (ref. ^21^), but it is now known to be associated with heterogeneous factors^22^. In line with the updated GOLD A–D classification of COPD^1^, Singh et al.^23^ have recently emphasized the importance of carefully assessing disease burden in individual patients rather than relying solely on spirometry data to plan for long-term COPD management^23^. Current best practice is for clinicians to use questionnaire-based tools to predict the progression of COPD^1^. The CCQ and the CAT are validated for assessing the COPD-specific health status, and the CCQ is useful in predicting mortality in patients with COPD^24^. Although the changes we found in CCQ and CAT scores were statistically significant, neither of these changes met the criteria for clinical important difference^10,11^.

In our study population, 80% of the patients had one or more chronic diseases in addition to COPD. COPD is increasingly recognized as a multicomponent disease with systemic consequences and effects on quality of life^25^, and it is not clear whether the CCQ and the CAT fully assess the impact of multimorbidity in this study. To improve the multidimensional evaluation of the progress of COPD, we therefore added clinically relevant and easily assessed outcomes. Specifically, the findings about hospital admissions and worsened exertion dyspnea strengthened our perception of disease progress.

Conducting and completing this type of pragmatic, real-life study about the patient outcomes of CME methods for GPs was an important achievement in itself as researchers and educators have called for studies on the clinical effectiveness of CME interventions^15^. CME research is particularly challenging to conduct because of the high demands for time and resources combined with challenges of real-life studies.

However, the pragmatic, real-life study design was primarily a strength as it enabled us to address outcomes in routine primary health care. Using short (2 × 2 h) CME sessions reflected the real-life CME situation for most GPs in Sweden today. Additionally, using self-reported data, such as symptoms, quality of life, and exacerbations gave first-hand information from patients on the effects of the COPD care provided by the participating GPs. Including several established outcome measures for COPD strengthened this study, as well as using new interesting outcomes, such as patients’ perceived information needs and care contacts within the interprofessional team.

This study evinced some of the limitations inherent in pragmatic trial design. Pragmatic studies are often subject to bias and confounding factors^26^, and a source of bias in PRIMAIR may have been the minor overlapping of the two pedagogical methods and teacher- or participant-related differences in teaching and learning. In contrast to strict, randomized controlled trials, we did not place constraints on patients, clinicians, or PHCCs during the study time. Although most of the GPs at the 24 PHCCs participated in the study at baseline, the GPs’ incentives and motivation to participate in the CME and/or complete the study may have varied. Additionally, PHCCs may have differed in patient-GP continuity and staffing. Furthermore, self-reported data are not recommended for use as the sole measure of guideline adherence^27^, as they entail a risk of misclassification bias (misunderstanding) and recall bias.

Cluster randomization of the PHCCs strengthened the results, as it reduced the likelihood of possible contamination across GPs at each PHCC (cluster). However, a limitation of the study was the absence of a reference arm (no CME to the GPs), which was a consequence of limited research resources. Another limitation was that we had 425 rather than the 460 patients who power calculation indicated we needed. These two limitations contributed to uncertainty about whether our results were associated with the CME or were a sign of the expected and inevitable progress of the disease. However, previous results of PRIMAIR showed that the GPs who participated in the CME sessions improved their levels of knowledge about COPD more than GPs without any CME^7^. We therefore reason that the two CME methods may have had equally positive yet modest effects on some of the patient outcomes during the 18-month observation time. It would have been interesting to investigate the reasons behind the limited effects on the patient outcomes we found. This was, however, not the aim of the study. Nonetheless, we consider our results as inherently interesting as the educational methods we have studied are routinely used for GPs’ CME.

As real-life studies are an important source of evidence about the effectiveness of educational methods^28^, the findings of this study increase knowledge about implementing CME in primary health care. This, in turn, may help educators and researchers conduct, assess, and make conclusions about CME programs that target the management of complex, chronic health issues, such as COPD. Using single CME interventions to study outcomes related to patients’ health will continue to be a challenge for educational and implementation researchers, yet research in this area should continue, as every project can contribute to the evidence base for future systematic reviews and meta-analyses^15^. Opportunities and time to attend evidence-based educational activities and collegial discussions should be the cornerstone of professional development of primary care clinicians. It is important to understand and regard professional development and CME as a long-term project, in which acquiring new knowledge and skills always is based on, and adds on, to what the individual knows before.

In summary, there were no differences between participatory (case method learning) and didactic (traditional lecture) CME methods for GPs, when the effectiveness of these methods was assessed by studying the changes in COPD patients’ health outcomes over 18 months. However, regardless of which of these CME types the GPs participated in, the prevalence of smoking decreased and use of pulmonary rehabilitation increased in the patients. CME’s primary value may thus lay in improving GPs’ adherence to guidelines, which should lead to long-term positive changes in patient health.

## Methods

We used the 2010 Consolidated Standards of Reporting Trials (CONSORT) statement: extension for cluster randomized trials^29^, the CONSORT checklist (Supplementary Table 1), and flow chart (Fig. 1). The present cluster randomized controlled trial^6^, including a model consent form and other related documentation given to participants, was approved by the Regional Ethical Review Board of Stockholm (ref 2013/232-31/5). Prior to patient enrollment, all PHCC managers and GPs provided written informed consent to be involved in the study. All patients provided written informed consent to participate in the study and they were allocated to the same study arm as their GPs. The study was registered at www.clinicaltrials.gov on 10 August 2014, Identifier NCT02213809. The first participant was enrolled 14 August 2014.

### Patient recruitment

The study enrollment for the parts of PRIMAIR that involved patients followed the cluster randomization of the patients’ PHCCs (Fig. 1). Both GPs and patients were recruited in 2014, after the research group first had computer randomized the 24 participating PHCCs (clusters) in Stockholm County, Sweden, to one of two study arms. The included PHCCs had the minimum of 10,000 registered patients and thus several GPs. In one of the arms, GPs were given CME (2 × 2 h) via case method learning (12 PHCCs) and in the other arm traditional lectures (12 PHCCs) were used. Apart from few exceptions, all the GPs on duty on the days of the CME sessions took part in the CME.

The research group identified all patients with a diagnosis of COPD in the PHCCs’ medical records. They then confirmed the diagnoses by applying GOLD 2013 guidelines^30^ to the spirometry results in patients’ electronic medical records. The guidelines defined COPD as the ratio of forced expiratory volume of one second and forced vital capacity (FEV1/FVC) < 0.70. Patients with FEV1, 30–79% of predicted, after bronchodilatation (GOLD stages 2 and 3), were included in the study. The sample size was determined by a power calculation based on the mean and standard deviation of the CCQ^8^ and the minimal clinically important difference (MCID) of 0.4 in the CCQ^10^. This resulted in the need for a minimum of 230 patients in each study arm. On the basis of earlier studies on cluster randomization in primary care^31–33^, we used the intraclass correlation coefficient of 0.01 in the power calculation. A total of 957 randomly selected patients were invited to participate, 40–45 from each PHCC.

### CME sessions for GPs

Five teachers, all GPs competent and experienced in COPD management, ran two 2-h sessions at each PHCC during 2014–2015. The first session was held after the baseline data from the patients were collected, and the other session a maximum of 3 months later. One teacher used case method learning at 12 PHCCs and four teachers used traditional lectures (each at 2–4 PHCCs). The learning outcomes (ILOs), activities, and assessments of the CME were aligned in accordance with John Biggs’^34^ theory of constructive alignment. The ILOs were derived from pre-2015 COPD guidelines^30,35,36^ and from a 2013 qualitative study that described GPs’ experiences of barriers to and facilitators of implementing COPD guidelines^37^. In short, the ILOs included knowledge about approaches to diagnostics, treatments (both pharmacological and non-pharmacological), and monitoring of patients with COPD in primary care settings^6^.

The case method learning activities were interactive rather than didactic. At a case method learning seminar, the teacher facilitated a discussion about one or two open-ended, real-life narratives (i.e., cases) that were written from a profession-specific perspective^38^. Participating in case method learning required previous knowledge and clinical experience in the subject. The primary aim of case method learning techniques was to improve clinical decision-making skills.

The traditional lectures were semi-didactic; that is, the lectures could include short patient case vignettes that may or may not have led to some interaction between the teacher and the participants. At these lectures, the teacher had the role of an academic expert. Traditional lectures primarily aimed to reduce knowledge barriers at the level of the individual participant.

### Data collection

Patient data were collected by mailing the patients the same questionnaire (Supplementary Methods) at baseline (2014) and at 18 months.

The primary outcome measure was the total CCQ score. The CCQ assesses disease-related health status, including airway symptoms, limitations in physical activity (functionality), and emotional dysfunction (mental health)^8^. The questions apply to the previous week. The CCQ uses a seven-point scale from 0 to 6, and total score is calculated as the mean of the sum of all the items. Higher values indicate worse health status; the MCID is 0.4 units^10^.

The secondary outcome measures included the total CAT score^9^, which assesses the impact of COPD symptoms on health status (scale 0–40; higher values indicate worse symptoms and health status, MCID = 2 points^11^); the total mMRC score^39^, which grades the impact of breathlessness on daily activities (scale 0–4; higher values indicate more dyspnea); and the total Lung Information Needs Questionnaire (LINQ) score^40^, which assesses patients’ perceived needs for information about COPD (scale 0–25, higher values indicate greater needs for information). Additional secondary outcomes were exacerbations, comorbidities, health care visits, smoking, treatments, and education levels. This information was gathered via the patient questionnaires.

A COPD exacerbation was defined as a patient-reported intermittent period of deterioration in the disease in the previous 6 months that had warranted an unscheduled or emergency visit to a PHCC or hospital and/or additional medication with antibiotics and/or oral steroids.

### Statistics

We performed the statistical analysis with STATA version 14 (ref. ^41^) and SPSS version 25 (ref. ^42^). We used SPSS to calculate summary statistics such as means, proportions, and confidence intervals with standard methods. In STATA, we used random-effects models with robust standard errors, xtreg for continuous variables, and xtlog for dichotomous variables to analyze the difference in differences (DiD) between the two CME methods over time. Both methods adjusted for clusters and for whether there was an asthma/COPD clinic at the PHCC. First, the difference in outcome between 18 months and baseline was estimated separately for the case method learning arm and the traditional lectures arm. Second, the DiD was estimated by applying the methods described above completed by margins. Margins are post-estimation statistics calculated from predictions of a previously fit model at fixed values of some covariates and averaging over the remaining covariates^43^. The contrast between the marginal effects of time in the two study arms was the average effect in the probability (dichotomous outcomes) or in scores (continuous outcomes).

The DiD of the outcome probabilities (or scores) was estimated using the margins. The result was the average difference in the probability (or in scores) of *y* between *x* = 0 (case method learning) and *x* = 1 (traditional lectures). The results are shown as Δ% for dichotomous outcomes or Δ score for continuous outcomes or *P* values of < 0.05 were considered statistically significant.

### Reporting summary

Further information on research design is available in the Nature Research Reporting Summary linked to this article.

## Supplementary information

Supplementary Information

Reporting Summary

## Data Availability

Data analyzed in this study are available from the corresponding author in response to requests that comply with ethical principles of good research.

## Code availability

SPSS and STATA syntax files can be requested by sending an e-mail to the corresponding author.
