# Supplementary material for: Patient outcomes following GPs’ educations about COPD: a cluster randomized controlled trial
Source: NPJ Prim Care Respir Med. 2020 Oct 15;30:44. doi: 10.1038/s41533-020-00204-w (PMC7566632; doi:10.1038/s41533-020-00204-w)
Supplement: Supplementary file 1 — Supplementary Information [file 41533_2020_204_MOESM1_ESM.pdf]

Supplementary Table 1: CONSORT 2010 checklist of information to include when reporting a cluster randomized trial

Section/Topic	Item No	Standard Checklist item	Extension for cluster designs	Page No *
**Title and abstract**				
	1a	Identification as a randomised trial in the title	Identification as a cluster randomised trial in the title	1
	1b	Structured summary of trial design, methods, results, and conclusions (for specific guidance see CONSORT for abstracts)[i,ii]	See table 2	2
**Introduction**				
**Background and objectives**	2a	Scientific background and explanation of rationale	Rationale for using a cluster design	2, 4
	2b	Specific objectives or hypotheses	Whether objectives pertain to the the cluster level, the individual participant level or both	5
**Methods**				
**Trial design**	3a	Description of trial design (such as parallel, factorial) including allocation ratio	Definition of cluster and description of how the design features apply to the clusters	2, 11-12
	3b	Important changes to methods after trial commencement (such as eligibility criteria), with reasons		NA
**Participants**	4a	Eligibility criteria for participants	Eligibility criteria for clusters	11-12
	4b	Settings and locations where the data were collected		11-12
**Interventions**	5	The interventions for each group with sufficient details to allow replication, including how and when they were actually administered	Whether interventions pertain to the cluster level, the individual participant level or both	11-13, Figure 1
**Outcomes**	6a	Completely defined pre-specified primary and secondary outcome measures, including how	Whether outcome measures pertain to the cluster level, the individual participant level or both	13-14

		and when they were assessed		
	6b	Any changes to trial outcomes after the trial commenced, with reasons		NA
**Sample size**	7a	How sample size was determined	Method of calculation, number of clusters(s) (and whether equal or unequal cluster sizes are assumed), cluster size, a coefficient of intracluster correlation (ICC or *k*), and an indication of its uncertainty	12
	7b	When applicable, explanation of any interim analyses and stopping guidelines		NA
**Randomisation:**				
**Sequence generation**	8a	Method used to generate the random allocation sequence		11
	8b	Type of randomisation; details of any restriction (such as blocking and block size)	Details of stratification or matching if used	NA
**Allocation concealment mechanism**	9	Mechanism used to implement the random allocation sequence (such as sequentially numbered containers), describing any steps taken to conceal the sequence until interventions were assigned	Specification that allocation was based on clusters rather than individuals and whether allocation concealment (if any) was at the cluster level, the individual participant level or both	2, 11, Figure 1
**Implementation**	10	Who generated the random allocation sequence, who enrolled participants, and who assigned participants to interventions	Replace by 10a, 10b and 10c	
	10a		Who generated the random allocation sequence, who enrolled clusters, and who assigned clusters to interventions	11
	10b		Mechanism by which individual participants were included in clusters for the purposes of the trial (such as complete enumeration, random sampling)	2, 11, Figure 1
	10c		From whom consent was sought (representatives of the cluster, or	15

			individual cluster members, or both), and whether consent was sought before or after randomisation	
**Blinding**	11a	If done, who was blinded after assignment to interventions (for example, participants, care providers, those assessing outcomes) and how		NA
	11b	If relevant, description of the similarity of interventions		12
**Statistical methods**	12a	Statistical methods used to compare groups for primary and secondary outcomes	How clustering was taken into account	2, 14
	12b	Methods for additional analyses, such as subgroup analyses and adjusted analyses		14
**Results**				
**Participant flow (a diagram is strongly recommended)**	13a	For each group, the numbers of participants who were randomly assigned, received intended treatment, and were analysed for the primary outcome	For each group, the numbers of clusters that were randomly assigned, received intended treatment, and were analysed for the primary outcome	Figure 1
	13b	For each group, losses and exclusions after randomisation, together with reasons	For each group, losses and exclusions for both clusters and individual cluster members	Figure 1
**Recruitment**	14a	Dates defining the periods of recruitment and follow-up		11, 13
	14b	Why the trial ended or was stopped		NA
**Baseline data**	15	A table showing baseline demographic and clinical characteristics for each group	Baseline characteristics for the individual and cluster levels as applicable for each group	Tables 1-3
**Numbers analysed**	16	For each group, number of participants (denominator) included in each analysis and whether the analysis	For each group, number of clusters included in each analysis	4, Figure 1

		was by original assigned groups		
**Outcomes and estimation**	17a	For each primary and secondary outcome, results for each group, and the estimated effect size and its precision (such as 95% confidence interval)	Results at the individual or cluster level as applicable and a coefficient of intracluster correlation (ICC or k) for each primary outcome	Tables 1-3<br><br>(ICC for each outcome NA)
	17b	For binary outcomes, presentation of both absolute and relative effect sizes is recommended		
**Ancillary analyses**	18	Results of any other analyses performed, including subgroup analyses and adjusted analyses, distinguishing pre-specified from exploratory		Tables 1-3
**Harms**	19	All important harms or unintended effects in each group (for specific guidance see CONSORT for harms[iii])		NA
**Discussion**				
**Limitations**	20	Trial limitations, addressing sources of potential bias, imprecision, and, if relevant, multiplicity of analyses		8-10
**Generalisability**	21	Generalisability (external validity, applicability) of the trial findings	Generalisability to clusters and/or individual participants (as relevant)	9
**Interpretation**	22	Interpretation consistent with results, balancing benefits and harms, and considering other relevant evidence		8-11
**Other information**				
**Registration**	23	Registration number and name of trial registry		2
**Protocol**	24	Where the full trial protocol can be accessed, if available		3, Reference 6
**Funding**	25	Sources of funding and other support (such as supply of drugs), role of funders		16

** Note: page numbers optional depending on journal requirements*

Supplementary Methods
The patient questionnaire.

Questions about your lung disease

1. **How old are you?** ……………

2. **At which health clinic/medical centre/doctor's practice is your doctor based?**

………………………………………………………………… ☐ Not applicable

3. **What lung disease do you have? Tick whichever option(s) you think apply.**

☐ COPD ☐ Asthma ☐ Chronic bronchitis ☐ Other:____________________ ☐ Don't know

☐ I have never had respirator problems ☐ I have no lung disease

4. **How old were you when your lung disease started to trouble you?**

☐ Under 30 ☐ 30-50 ☐ 51-60 ☐ 61-70 ☐ Over 70

5. Do you have/have you ever had the following diseases?

(Tick the appropriate box(es)).

☐ Diabetes ☐ Heart disease ☐ Stroke ☐ Hypertension ☐ Anxiety/Depression

☐ Sleep apnoea ☐ Rheumatic disease ☐ Cancer ☐ Heartburn

☐ Chronic pain ☐ None of these

6. **Have you ever been medically diagnosed with asthma?**

☐ Yes ☐ No ☐ Don't know

Questions about your medication

**7.**	**Have you taken fast-acting bronchial dilator such as Bricanyl, Ventoline, Airomir, Airsalb, Salbutamol, Ventilastin or Buventol in the *past week* for respiratory problems?**<br><br>☐ Yes ☐ No ☐ Don't know
**8.**	**Have you used a cortisone inhalant such as Pulmicort, Flutide, Becotide, Asmanex, Giona, Novopulmon, Budesonid, Aerobec, Alvesco or Beclomet in the *past six months*?**<br><br>☐ Regularly ☐ Occasionally ☐ No ☐ Don't know
**9.**	**Have you used the bronchial dilator Atrovent in the *past six months*?**<br><br>☐ Regularly ☐ Occasionally ☐ Once or twice ☐ No ☐ Don't know
**10.**	**Have you used any of the bronchial dilators Spiriva, Eklira or Seebri in the *past six months*?**<br><br>☐ Regularly ☐ Occasionally ☐ No ☐ Don't know
**11.**	**Have you used the long-acting bronchial dilators such as Oxis, Serevent, Formatris or Onbrez in the *past six months*?**<br><br>☐ Regularly ☐ Occasionally ☐ No ☐ Don't know
**12.**	**Have you taken *extra doses* of long-acting bronchial dilators such as Oxis, Serevent, Formatris or Onbrez in the *past week*?**<br><br>☐ Yes ☐ No ☐ Don't know
**13.**	**Have you used Symbicort, Seretide, Airflusal, Relanio, Bufomix, Flutiform or Innovair (combination of long-acting bronchial dilator and cortisone) in the *past six months*?**<br><br>☐ Regularly ☐ Occasionally ☐ No ☐ Don't know

**14.**	**Have you taken *extra doses* of Symbicort, Seretide, Airflusal, Relanio, Bufomix, Flutiform or Innovair in the past week?**<br><br>☐ Yes ☐ No ☐ Don't know
**15.**	**Have you used Daxas in the *past six months*?**<br><br>☐ Regularly ☐ Occasionally ☐ No ☐ Don't know
**16.**	**Have you needed to take *cortisone tablets* (Betapred or Prednisolon) on account of a deterioration in your lung disease in the *past six months*?**<br><br>☐ Yes, once ☐ Yes, on two separate occasions ☐ Yes, on more than two separate occasions<br>☐ I take cortisone tablets regularly ☐ No
**17.**	**Have you needed to take *antibiotics* on account of a deterioration in your lung disease at any time in the *past six months*?**<br><br>☐ Yes, once ☐ Yes, on two separate occasions ☐ Yes, on more than two separate occasions ☐ No
**18.**	**Bearing in mind how you use your respiratory medication and what your doctor or asthma/COPD nurse has recommended, which statement applies the closest to you?**<br><br>☐ I always take what's recommended ☐ I usually take what's recommended<br>☐ I sometimes take what's recommended ☐ I rarely take what's recommended<br>☐ I never take what's recommended ☐ I don't take respiratory medication
**19.**	**Have you been prescribed medicine for preventing or treating osteoporosis in *the past year*?**<br><br>☐ Yes ☐ No ☐ Don't know

Questions about the problems your lung disease causes you

**20.**	**Have you been woken at night by coughing, wheezing or respiratory difficulties in *the past week*?**<br><br>☐ Yes, once ☐ Yes, several times ☐ No
**21.**	**Have you made an emergency appointment with your doctor/medical centre on account of a deterioration in your lung disease in the *past six months*?**<br><br>☐ Yes, once ☐ Yes, twice ☐ Yes, more than twice ☐ No
**22.**	**Have you sought emergency help from a hospital on account of a deterioration in your lung disease in the *past six months*?**<br><br>☐ Yes, once ☐ Yes, twice ☐ Yes, more than twice ☐ No
**23.**	**Have you been in hospital on account of your lung disease in the *past six months*?**<br><br>☐ Yes, once ☐ Yes, twice ☐ Yes, more than twice ☐ No
**24.**	**Have you sought any kind of emergency medical help on account of a deterioration in your lung disease in t*he past year*?**<br><br>☐ Yes, once ☐ Yes, twice ☐ Yes, three times ☐ Yes, more than three times ☐ No
**25.**	**How would you describe the severity of your lung disease?**<br><br>☐ Very mild ☐ Mild ☐ Moderately severe ☐ Severe ☐ Very severe<br><br>☐ I have no lung disease
**26.**	**How often and when do you suffer from breathlessness? Tick whichever option(s) you think apply:**<br>☐ When I really exert myself, not when I go for a quick walk or walk uphill.<br>☐ When I go for a quick walk or walk uphill.<br>☐ When I walk on level ground at the same pace as another person of my own age.<br>☐ I get so breathless when I walk on level ground that I have to stop despite going at my own pace.<br>☐ When I wash or dress myself.

Questions about your dealings with the medical services

27. **Where do you normally go to have your lung disease checked? Tick the box(es):**

☐ My local GP ☐ My occupational physician

☐ The hospital (pulmonary or medical clinic) ☐ A private pulmonologist

☐ An asthma/COPD nurse ☐ Other ☐ Nowhere

☐ I have no lung disease (go to question 42)

28. **Do you know which doctor is responsible for treating your lung disease?**

☐ Yes ☐ No

29. Have you visited an asthma/COPD nurse on account of your lung disease in the past year? (NOT emergency visits)

☐ Yes ☐ No ☐ Don't know

30. Have you been to see a physiotherapist on account of your lung disease in the past year?

☐ Yes ☐ No ☐ Don't know

31. Have you been to see an occupational therapist on account of your lung disease in the past year?

☐ Yes ☐ No ☐ Don't know

32. Have you been to see a dietician on account of your lung disease in the past year?

☐ Yes ☐ No ☐ Don't know

33. Have you been to see a counsellor on account of your lung disease in the past year?

☐ Yes ☐ No ☐ Don't know

34. Do you think you are suitably informed about how to handle a deterioration in your lung disease?

☐ Yes ☐ Yes, somewhat ☐ Yes, a little ☐ No

**35.**	**Have you been given a pneumococcal vaccine in the past 5 years?**<br><br>☐ Yes ☐ No ☐ Don't know
**36.**	**Have you been given an influenza vaccine in the past 12 months?**<br><br>☐ Yes ☐ No ☐ Don't know

Other questions

**37.**	**What is the *highest* level of education you have achieved?**<br><br>☐ Less than 5 years in school<br>☐ Primary school<br>☐ Grammar school or the equivalent<br>☐ 2-year upper secondary/vocational college<br>☐ 3/4-year upper secondary<br>☐ University or university college, up to 2.5 years<br>☐ University or university college, 3 years or more
**38.**	**How tall are you?** ……………… cm
**39.**	**How much do you weigh?** ……………kg
**40.**	**If you smoke/have smoked: for how many years have you been/were you a daily smoker?** …………
**41.**	**If you smoke/have smoked: How many cigarettes do/did you smoke a day on average?**.........................

42. **Answer this question if you smoke or stopped smoking in the past 5 years.**

Have you *been offered* professional medical help to quit smoking?

☐ Yes ☐ No ☐ Don't know

43. **Answer this question if you smoke or stopped smoking in the past 5 years.**

Have you *been given* professional medical help to quit smoking, either individually or in a group?

☐ Yes ☐ No ☐ Don't know

44. **Answer this question if you smoke/have smoked.**

Have you used drugs, OTC or prescription, in order to quit smoking?

☐ Yes, nicotine replacement (e.g. patches or chewing gum) ☐ Pills to reduce craving (Zyban or Champix)

☐ Other ……………………………………………☐ No

[After question 44, two attached questionnaires; the Lung Information Needs Questionnaire (LINQ) and the COPD Assessment Test (CAT)]
