# Supplementary material for: Patient outcomes following GPs’ educations about COPD: a cluster randomized controlled trial
Source: NPJ Prim Care Respir Med. 2020 Oct 15;30:44. doi: 10.1038/s41533-020-00204-w (PMC7566632; doi:10.1038/s41533-020-00204-w)
Supplement: Supplementary file 2 — Reporting Summary [file 41533_2020_204_MOESM2_ESM.pdf]

Corresponding author(s): NPJPCRM-01035 Hanna Sandelowsky

Last updated by author(s): Sep 12, 2020

Reporting Summary

Nature Research wishes to improve the reproducibility of the work that we publish. This form provides structure for consistency and transparency in reporting. For further information on Nature Research policies, see our Editorial Policies and the Editorial Policy Checklist.

Statistics

For all statistical analyses, confirm that the following items are present in the figure legend, table legend, main text, or Methods section.

n/a	Confirmed	
☐	☒	The exact sample size (*n*) for each experimental group/condition, given as a discrete number and unit of measurement
☐	☒	A statement on whether measurements were taken from distinct samples or whether the same sample was measured repeatedly
☐	☒	The statistical test(s) used AND whether they are one- or two-sided<br>*Only common tests should be described solely by name; describe more complex techniques in the Methods section.*
☐	☒	A description of all covariates tested
☐	☒	A description of any assumptions or corrections, such as tests of normality and adjustment for multiple comparisons
☐	☒	A full description of the statistical parameters including central tendency (e.g. means) or other basic estimates (e.g. regression coefficient) AND variation (e.g. standard deviation) or associated estimates of uncertainty (e.g. confidence intervals)
☐	☒	For null hypothesis testing, the test statistic (e.g. *F*, *t*, *r*) with confidence intervals, effect sizes, degrees of freedom and *P* value noted<br>*Give P values as exact values whenever suitable.*
☒	☐	For Bayesian analysis, information on the choice of priors and Markov chain Monte Carlo settings
☒	☐	For hierarchical and complex designs, identification of the appropriate level for tests and full reporting of outcomes
☐	☒	Estimates of effect sizes (e.g. Cohen's *d*, Pearson's *r*), indicating how they were calculated

Our web collection on statistics for biologists contains articles on many of the points above.

Software and code

Policy information about availability of computer code

Data collection	We collected the data in this pragmatic, cluster ransomized controlled trial in Stockholm, Sweden (2014-2017) first by questionnaires (paper format), then transferred them to digital files using Microsoft Office functions.
Data analysis	We performed the statistical analysis with STATA version 14 (41) and SPSS version 25. The programs are available on request.

For manuscripts utilizing custom algorithms or software that are central to the research but not yet described in published literature, software must be made available to editors and reviewers. We strongly encourage code deposition in a community repository (e.g. GitHub). See the Nature Research guidelines for submitting code & software for further information.

Data

Policy information about availability of data

All manuscripts must include a data availability statement. This statement should provide the following information, where applicable:

- Accession codes, unique identifiers, or web links for publicly available datasets
- A list of figures that have associated raw data
- A description of any restrictions on data availability

Data analyzed in this study are available from the corresponding author in response to requests that comply with ethical principles of good research.

Field-specific reporting

Please select the one below that is the best fit for your research. If you are not sure, read the appropriate sections before making your selection.

☒ Life sciences ☐ Behavioural & social sciences ☐ Ecological, evolutionary & environmental sciences

For a reference copy of the document with all sections, see nature.com/documents/nr-reporting-summary-flat.pdf

Life sciences study design

All studies must disclose on these points even when the disclosure is negative.

Sample size

The sample size was determined by a power calculation based on the mean and standard deviation of the Clinical COPD Questionnaire (CCQ) and the minimal clinically important difference of 0.4 in the CCQ . This resulted in the need for a minimum of 230 patients in each study arm. On the basis of earlier studies on cluster randomization in primary care, we used the intraclass correlation coefficient of 0.01 in the power calculation. A total of 957 randomly selected patients were invited to participate, 40 to 45 from each primary health care center (PHCC).

Data exclusions

At baseline, 542 patients completed the questionnaire (response rate 57%), and at 18 months, 425 replied to the follow-up questionnaire (final response rate 44%). The 117 patients who did not complete the study at 18 months were excluded.

Replication

The findings were not replicated due to the pragmatic (real-world) nature of the study.

Randomization

The study enrollment for the patients followed the cluster randomization of the patients' primary health PHCC/general practitioners (GP). Both GPs and patients were recruited in 2014, after the research group first had computer randomized the 24 participating PHCCs (clusters) in Stockholm County, Sweden, to one of two study arms.

Blinding

Blinding was not possible; both investigators and participating GPs were aware of which type of educational session they attended. However, the population that was studied in this paper was the patients of the GPs. They might not have been aware of which type of ieducational ntervention their GPs had attended, but this issue was not registered or communicated, due to the pragmatic (real-world) nature of the study.

Reporting for specific materials, systems and methods

We require information from authors about some types of materials, experimental systems and methods used in many studies. Here, indicate whether each material, system or method listed is relevant to your study. If you are not sure if a list item applies to your research, read the appropriate section before selecting a response.

Materials & experimental systems

n/a	Involved in the study	
☒	☐	Antibodies
☒	☐	Eukaryotic cell lines
☒	☐	Palaeontology and archaeology
☒	☐	Animals and other organisms
☐	☒	Human research participants
☐	☒	Clinical data
☒	☐	Dual use research of concern

Methods

n/a	Involved in the study	
☒	☐	ChIP-seq
☒	☐	Flow cytometry
☒	☐	MRI-based neuroimaging

Human research participants

Policy information about studies involving human research participants

Population characteristics

Patients attending 24 PHCCs in Stockholm County (Sweden), with a diagnosis of COPD in GOLD stages 2 or 3, by spirometry, in the PHCCs' medical records.

Recruitment

Patients were recruited by mailed invitations in 2014, after their PHCCs had been recruited by email, and included in the study. The research group first had computer randomized the 24 participating PHCCs (clusters) in Stockholm County, Sweden, to one of two study arms. The included PHCCs had the minimum of 10.000 registered patients and thus several GPs.

Ethics oversight

The present cluster randomized controlled trial, including a model consent form and other related documentation given to participants, was approved by the Regional Ethical Review Board of Stockholm (ref 2013/232-31/5).

Note that full information on the approval of the study protocol must also be provided in the manuscript.

Clinical data

Policy information about clinical studies

All manuscripts should comply with the ICMJE guidelines for publication of clinical research and a completed CONSORT checklist must be included with all submissions.

Clinical trial registration	www.clinicaltrials.gov 10 August 2014, Identifier NCT02213809
Study protocol	Sandelowsky H, Krakau I, Modin S, Stallberg B, Nager A. Case Method in COPD education for primary care physicians: study protocol for a cluster randomised controlled trial. Trials. 2017;18(1):197. doi: 10.1186/s13063-017-1889-4
Data collection	We collected the data in this pragmatic, cluster ransomized controlled trial in Stockholm, Sweden (2014-2017) first by mailed questionnaires (paper format), then transferred the data to digital files using Microsoft Office functions. The data was password secured and storaged in locked facilities.
Outcomes	The primary outcome measure was the total CCQ score (0-6 points). The CCQ assesses disease-related health status, including airway symptoms, limitations in physical activity (functionality), and emotional dysfunction (mental health). The questions apply to the previous week. The CCQ uses a seven-point scale from zero to six, and total score is calculated as the mean of the sum of all the items. Higher values indicate worse health status; the minimal clinically important difference (MCID) is 0.4 units.<br>The secondary outcome measures included the total COPD Assessment Test (CAT) score, which assesses the impact of COPD symptoms on health status (scale 0-40; higher values indicate worse symptoms and health status, MCID = 2 points (11)); the total modified Medical Research Council dyspnea scale (mMRC) score, which grades the impact of breathlessness on daily activities (scale 0-4; higher values indicate more dyspnea); and the total Lung Information Needs Questionnaire (LINQ) score, which assesses patients' perceived needs for information about COPD (scale 0-25, higher values indicate greater needs for information). Additional secondary outcomes were exacerbations, comorbidities, health care visits, smoking, treatments, and education levels.
